# Ammonia-Oxidizing Archaea Show More Distinct Biogeographic Distribution Patterns than Ammonia-Oxidizing Bacteria across the Black Soil Zone of Northeast China

**DOI:** 10.3389/fmicb.2018.00171

**Published:** 2018-02-09

**Authors:** Junjie Liu, Zhenhua Yu, Qin Yao, Yueyu Sui, Yu Shi, Haiyan Chu, Caixian Tang, Ashley E. Franks, Jian Jin, Xiaobing Liu, Guanghua Wang

**Affiliations:** ^1^Key Laboratory of Mollisols Agroecology, Northeast Institute of Geography and Agroecology, Chinese Academy of Sciences, Harbin, China; ^2^State Key Laboratory of Soil and Sustainable Agriculture, Institute of Soil Science, Chinese Academy of Sciences, Nanjing, China; ^3^Department of Animal, Plant and Soil Sciences, AgriBio Centre for AgriBiosciences, La Trobe University, Bundoora VIC, Australia; ^4^Department of Physiology, Anatomy and Microbiology, La Trobe University, Bundoora VIC, Australia

**Keywords:** 454 pyrosequencing, ammonia oxidizers, biogeographic distribution, potential nitrification rate, *amoA* gene, Mollisols

## Abstract

Black soils (Mollisols) of northeast China are highly productive and agriculturally important for food production. Ammonia-oxidizing microbes play an important role in N cycling in the black soils. However, the information related to the composition and distribution of ammonia-oxidizing microbes in the black soils has not yet been addressed. In this study, we used the *amoA* gene to quantify the abundance and community composition of ammonia-oxidizing archaea (AOA) and ammonia-oxidizing bacteria (AOB) across the black soil zone. The *amoA* abundance of AOA was remarkably larger than that of AOB, with ratios of AOA/AOB in the range from 3.1 to 91.0 across all soil samples. The abundance of AOA *amoA* was positively correlated with total soil C content (*p* < 0.001) but not with soil pH (*p* > 0.05). In contrast, the abundance of AOB *amoA* positively correlated with soil pH (*p* = 0.009) but not with total soil C. Alpha diversity of AOA did not correlate with any soil parameter, however, alpha diversity of AOB was affected by multiple soil factors, such as soil pH, total P, N, and C, available K content, and soil water content. Canonical correspondence analysis indicated that the AOA community was mainly affected by the sampling latitude, followed by soil pH, total P and C; while the AOB community was mainly determined by soil pH, as well as total P, C and N, water content, and sampling latitude, which highlighted that the AOA community was more geographically distributed in the black soil zone of northeast China than AOB community. In addition, the pairwise analyses showed that the potential nitrification rate (PNR) was not correlated with alpha diversity but weakly positively with the abundance of the AOA community (*p* = 0.048), whereas PNR significantly correlated positively with the richness (*p* = 0.003), diversity (*p* = 0.001) and abundance (*p* < 0.001) of the AOB community, which suggested that AOB community might make a greater contribution to nitrification than AOA community in the black soils when ammonium is readily available.

## Introduction

Black soils, classified as dark Chernozems and also referred to Mollisols, are highly fertile and produce large agricultural yields in China and other countries (Liu et al., 2012). Black soils are globally distributed in four major regions: in central North America across the central plains of the United States and southern Canada, in central Asia of northeast China, in southeastern Europe across Russia and Ukraine, and in the Pampas of South America occupying most of central-eastern Argentina and Uruguay (Liu et al., 2012). In northeast China, the black soils are mainly distributed in a long and narrow area called the black soil zone which covers approximately 900 km from the north to south and 300 km from the east to west across Heilongjiang, Jilin and Liaoning provinces. Annual average temperature decreases and soil chemical fertility generally increases latitudinally from south to north of the black soil zone (Zhang et al., 2007), creating an ideal region for studying biogeographic distribution of microorganisms.

Our previous studies have revealed distinct biogeographic distribution of the bacterial and fungal communities across the black soil zone of China (Liu et al., 2014, 2015). The two overarching soil factors in determining the distribution of bacterial and fungal communities in this soil zone are soil pH and soil carbon content, respectively (Liu et al., 2014, 2015). Acidobacterial community, a major component of the soil bacteria, does not show distinct geographic distribution and its distribution pattern was predominately driven by soil pH (Liu et al., 2016). However, the distribution of functional microbial groups such as ammonia oxidizers and their related driving forces in this region have not been studied.

Ammonia oxidation is the first and often rate-limiting step of nitrification with major implication for the global N cycle (Kowalchuk and Stephen, 2001). This step is performed by two types of microbes: ammonia-oxidizing bacteria (AOB), belonging to two monophyletic groups within the beta- and gamma-proteobacteria (Purkhold et al., 2000), and ammonia-oxidizing archaea (AOA), belonging to *Thaumarchaeota* phylum (Brochier-Armanet et al., 2008). AOA and AOB co-exist in most agricultural soils and performed different ways in response to environmental disturbances (i.e., niche specialization) and resources utilization (i.e., niche differentiation) (Valentine, 2007; Erguder et al., 2009; Prosser and Nicol, 2012; Ouyang et al., 2016). As a member of AOB, Nitrospira have recently been proved to play important roles in comammox process of complete ammonia oxidation to nitrate (Daims et al., 2015), but their performance in agricultural soils remains unclear (Ouyang et al., 2017). Site-specific studies on the assessment of abundances and community compositions of ammonia oxidizers, and relative contribution of AOA and AOB to nitrification were well documented (He et al., 2007; Jia and Conrad, 2009; Ke et al., 2013; Ouyang et al., 2016). Most findings of these studies indicated that no single soil parameter (with the possible exception of soil pH) could explain the abundance and diversity of AOA and AOB (Prosser and Nicol, 2012). Recently, several large-scale investigations of the AOA or/and AOB in various environments, including forest soils (Stempfhuber et al., 2014), drylands (Hu et al., 2013), grasslands (Yao et al., 2013), and agricultural upland soils (Gubry-Rangin et al., 2011; Hu et al., 2014), as well as paddy soils (Hu et al., 2015) indicated that ammonia oxidizers are biogeographically distributed but the geographic distance on the explanation of community variation differed between AOA and AOB (Hu et al., 2013, 2014, 2015; Yao et al., 2013). Generally, soil pH was detected as the most dominating soil factor in niche specialization of AOA and AOB community (Gubry-Rangin et al., 2011; Hu et al., 2013, 2014, 2015); while other studies indicated that the distribution of AOB and AOA community was driven by multiple soil factors together (Prosser and Nicol, 2012; Yao et al., 2013).

In this study, the same soil samples for the previous study of bacterial and fungal community across the black soil zone were used; the abundances and community compositions of AOA and AOB were examined by targeting ammonia monooxygenase (*amoA*) gene using the real-time qPCR and 454 pyrosequencing method, respectively. In addition, the potential nitrification rate (PNR) of all soil samples was determined and the relationship between PNR and abundance and composition of AOA and AOB was analyzed. Specifically, this study aimed to address the following questions: (1) What are the distribution patterns of ammonia-oxidizing microbial communities across the black soil zone? (2) Which ammonia-oxidizing microbes, AOA or AOB are highly correlated with PNR in the black soils? (3) What factors drive AOA or AOB community distribution in the black soils?

## Materials and methods

### Soil sampling and DNA extraction

The methods of soil sampling and the determinations of soil physicochemical properties were described previously (Liu et al., 2014). Briefly, based on the database of China Black Soil Ecology (http://www.blackland.csdb.cn), the plow layer of 26 soil samples were collected from arable lands across the black soil zone of northeast China in September 2012 (Figure S1). Water content and other soil physicochemical properties were measured immediately after soil samples collected. Soil DNA was extracted from soil samples (0.5 g wet weight) with the E.Z.N.A Soil DNA (OMEGA, USA), according to the manufacturer's instruction. The extracted DNA was diluted in TE buffer (10 mM Tris-HCl, 1 mM EDTA, pH 7.0) and stored at −20°C until use. The soil physicochemical properties are shown in Table S1.

### Measurement of potential nitrification rates (PNR)

PNR was measured by the chlorate inhibition shaken soil-slurry method with modifications (Kurola et al., 2005). Briefly, 5 g fresh soil was added to an Erlenmeyer flask with 20 ml of phosphate buffer solution (g l^−1^: NaCl, 8.0; KCl, 0.2; Na_2_HPO_4_, 0.2; NaH_2_PO_4_, 0.2), with a final concentration of 1 mM (NH_4_)_2_SO_4_, and pH adjusted to 7.1. In order to inhibit nitrite oxidation, potassium chlorate was added to the tubes at a final concentration of 10 mM. The soil slurry was then incubated at 25°C for 24 h in the dark, and nitrite was extracted with 5 ml of 2 M KCl solution. Aliquots of 5 ml were subsequently removed using a wide-mouth pipette at 2, 8, 14 and 24 h after the start of the incubation. After centrifugation, the supernatant was filtered and stored at −20°C until analysis. The ${\text{NO}}_{2}^{-}$-N concentrations were measured using a flow injection autoanalyzer (SKALAR, San++, Netherlands). PNR was calculated from the rate of the linear accumulation in nitrite concentrations during the incubation (μg ${\text{NO}}_{2}^{-}$-N g^−1^ h^−1^).

### Quantitative PCR analysis

The abundances of AOA and AOB ammonia monooxygenase gene *amoA* were quantified by real-time PCR using the primer pairs Arch-amoAF/Arch-amoAR (Francis et al., 2005) and amoA1F/amoA2R (Rotthauwe et al., 1997), respectively. To make the AOA and AOB *amoA* gene standard, the *amoA* gene was PCR-amplified from extracted DNA with the primers pairs Arch-amoAF/Arch-amoAR and amoA1F/amoA2R, respectively. Purified DNA was cloned into pMD18-T plasmid vector (TaKaRa, Dalian, China) and transformed into competent cells of *Escherichia coli* DH5α. The positive clones containing a proper insert of the target gene were selected to extract plasmid. The plasmid DNA concentration was determined on a Nanodrop 2000 (Thermo Scientific, USA), and the copy numbers of *amoA* genes were calculated with the concentration of the extracted plasmid DNA. The standard curves were generated using 10-fold serial dilutions of a plasmid containing the AOA or AOB target gene inserts. Each PCR reaction contained 10 μl of SYBR Premix Ex Taq™ (Takara, Dalian, China), 0.4 μl of 10-μM forward and reverse primers (each), 7.2 μl of sterilized MilliQ water, and 2 μl of standard or extracted soil DNA. The PCR was performed in a LightCycler® 480 (Roche Applied Science) as follows: 95°C for 1 min, followed by 30 cycles of 10 s at 95°C, 30 s at 53°C for AOA and 55°C for AOB, 1 min at 72°C and follow by a plate read at 83°C. First, the copy number of AOA and AOB genes was calculated using a regression equation for converting the cycle threshold (Ct) value to the known number of copies in the standards. Then the copy number of AOA and AOB genes were converted into per gram of dry soil by the following formula: Copy number $=\frac{\left(\frac{\text{X}}{\text{n}}\right)\times \text{ C }\times \text{ V}}{0.5\times \left(1-\text{M}\right)}$, where X indicates the copy numbers of *amoA* gene detected by qPCR; n indicates the amount of DNA used as template in the qPCR (ng); C and V indicates the concentration (ng μl^−1^) and the volume (μl) of the extracted DNA, respectively; 0.5 indicates the amount of soil used for DNA extraction (g); M indicates soil moisture (Sun et al., 2015). For all assays, amplification efficiency ranged between 90 and 99%, and *R*^2^-values was 0.995–0.999. All of the real-time PCR reactions were run in triplicate for each soil sample.

### Bar-coded pyrosequencing

Pyrosequencing of the AOA and AOB *amoA* gene was performed using the primer pairs Arch-amoAF/Arch-amoAR (Francis et al., 2005) and amoA1F/amoA2R (Rotthauwe et al., 1997), respectively. The forward primer was modified to contain a unique 8 nt barcode at the 5′ end, and the reverse primer had the adaptor B to the 3′ end. Each sample was PCR amplified in a 25-μl reactions containing 10 ng of DNA under the following conditions: initial denaturation at 95°C for 2 min, followed by 35 cycles consisting of denaturation at 95°C for 30 s, annealing for 1 min at 53°C for AOA and 58°C for AOB, and extension at 72°C for 1 min, with a final extension at 72°C for 5 min. Each sample was amplified in triplicate, and the PCR products were pooled and purified using an Agarose Gel DNA purification kit (TaKaRa, Dalian, China). An equimolar amount of the PCR products were combined into one pooled sample and run on a Roche FLX 454 pyrosequencing machine at Personal Biotechnology Co., Ltd., Shanghai, China.

### Bioinformatics and statistics

Data were processed and analyzed using QIIME Pipeline Version 1.8.0 (http://qiime.sourceforge.net; Caporaso et al., 2010a). In brief, reads which were <350 bp in length or with a mean quality score <25 were removed, and the 8 bp barcode was examined to assign sequences to each sample. The trimmed sequences were chimera-detected and removed using the Uchime algorithm (Edgar et al., 2011). Clustering of high-quality sequences into operational taxonomic units (OTUs) was performed with UPARSE pipeline (http://drive5.com/uparse/) at 85% similarity level (Purkhold et al., 2000; Pester et al., 2012). A representative sequence from each phylotype was aligned using the Python Nearest Alignment Space Termination (PyNAST) tool (DeSantis et al., 2006; Caporaso et al., 2010b) with a relaxed neighbor-joining tree built using FastTree (Price et al., 2009). The taxonomic information of *amoA* gene sequences was determined using the ARB databases for AOA and AOB created by Abell et al. (2012). Furthermore, the subclusters of *Nitrososphaera* among AOA community was determined based on the taxonomic information proposed by Pester et al. (2012). To correct for differences in the sequencing efforts, a subset of 4900 and 4100 sequences for AOA and AOB was randomly selected per sample, respectively, and used for alpha-diversity and beta-diversity analyses. Alpha diversity was estimated by calculating the operational taxonomic unit (OTU) richness at the 85% sequence identity (Purkhold et al., 2000; Pester et al., 2012; Hu et al., 2015), and the phylogenetic diversity (PD) index (Faith, 1992). The pyrosequencing reads of the AOA and AOB *amoA* genes were submitted in the National Centre for Biotechnology Information (NCBI) Sequence Read Archive (SRA) with accession number SRP117126.

### Statistical analysis

Spearman's correlation coefficients were used to reveal significant correlations between the abundances of AOA and AOB, the diversity, the relative abundance of the taxonomic ammonia-oxidizing lineages, geochemical properties, and PNR using SPSS 18.0 for Windows. Phylogenetic identities of the representative sequences obtained in this study with those taxonomy-determined reference sequences from the NCBI database were aligned using ClustalX 1.81, and the neighbor-joining tree was constructed by Molecular Evolutionary Genetic Analysis software (MEGA 6.0) with 1000-fold bootstrap support (Tamuka et al., 2013). Pairwise UniFrac distances calculated for the total community analyses were visualized using non-metric multidimensional scaling (NMDS) plots as implemented in PRIMER v6 (Clarke and Warwick, 2001). All other statistical tests were performed in R software (v.2.8.1) (R Development Core Team, 2010). The significant effects of environmental variables on both AOA and AOB β-diversity patterns between the sites were conducted by the Mantel test analysis. Meanwhile, the partial Mantel Test was used to explain the correlations between *amoA* community composition and the soil factors and spatial factors (Shi et al., 2016). Canonical correspondence analysis (CCA) was performed to identify spatial factors and environmental variables in driving the difference in AOA and AOB community compositions with sampling latitude, pH, total C, N and P, C/N, available K and P, ${\text{NH}}_{4}^{+}$-N, ${\text{NO}}_{3}^{-}$-N and soil water content as the explanatory factors. Only the factors significantly corrected with *amoA* community composition were retained for the CCA analysis. A variance partitioning analysis (VPA) based on a redundancy analysis procedure was conducted to quantify the relative contributions of environmental variables and special factors on the AOA and AOB community compositions using the “vegan” package in the R environment.

## Results

### Abundance of AOA and AOB, and potential nitrification rates

The abundance of AOA *amoA* of all soil samples ranged from 2.51 × 10^6^ to 3.88 × 10^7^ copies per gram of dry soil, with the lowest and the highest values observed in the locations GZL and BA, respectively (Table S1). The abundance of AOB *amoA* varied from 2.92 × 10^4^ to 2.19 × 10^6^ copies per gram of dry soil, with the lowest and the highest numbers found in the locations GZL and BY, respectively (Table S1). The *amoA* abundance of AOA was consistently larger than that of AOB, resulting in AOA/AOB *amoA* gene ratio ranged from 3.1 in the location BQ to 91.0 in the location DH1 (Table S1). Pairwise regression analysis indicated that the abundance of AOA *amoA* correlated highly with soil total C content (*r* = 0.830, *p* < 0.0001) but not with soil pH (*r* = 0.312, *p* = 0.120). On the contrary, the abundance of AOB *amoA* positively correlated with soil pH (*r* = 0.502, *p* = 0.0009) but not with soil total C content (*r* = 0.170, *p* = 0.408) (Figure S2).

**Figure 1:**
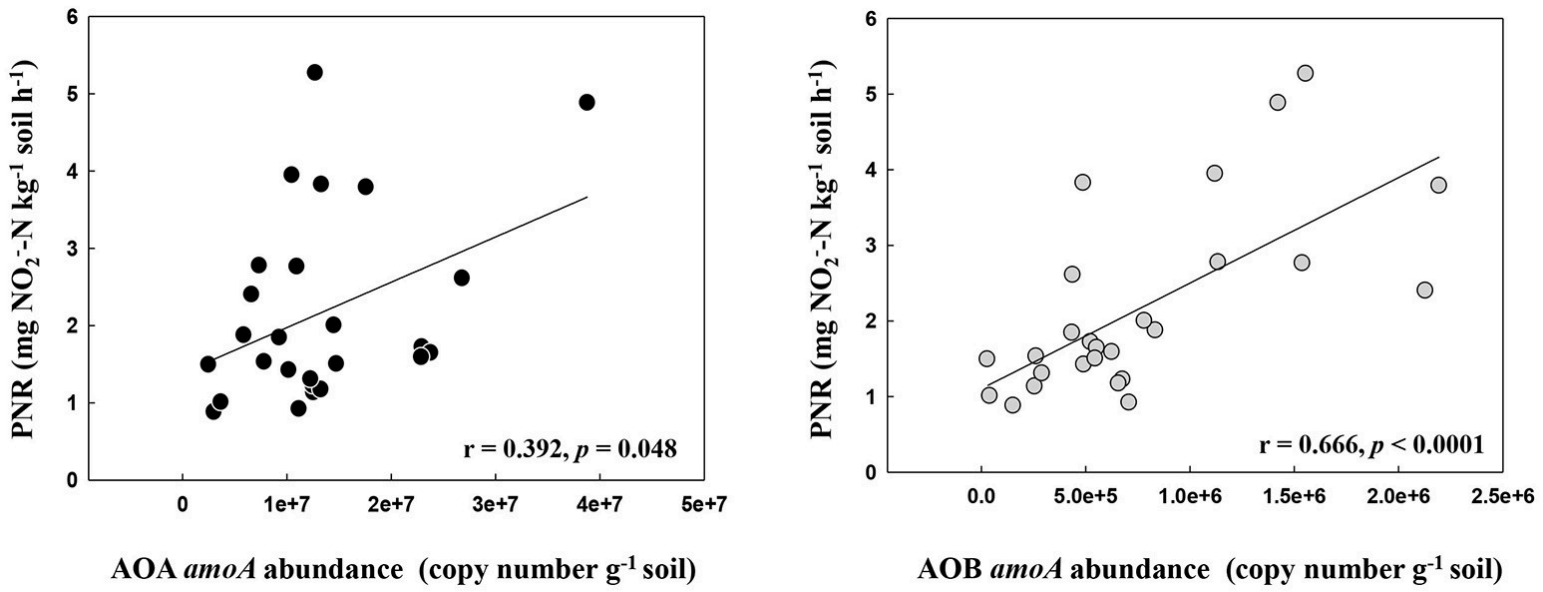
The relationship between AOA and AOB *amoA* gene abundance and potential nitrification rates in the black soils.

The PNR of all samples ranged from 0.88 to 5.27 mg ${\text{NO}}_{2}^{-}$-N kg^−1^ dry soil h^−1^, with the lowest and the highest values determined in the locations DH1 and HRB, respectively (Table S1). Pairwise regression analysis revealed that PNR weakly correlated with the abundance of AOA (*r* = 0.392, *p* = 0.048) but correlated highly with the abundance of AOB (*r* = 0.666, *p* < 0.0001) (Figure 1).

### Taxonomic classification of AOA and AOB

In total, 417,205 quality AOA *amoA* sequences were obtained from all 26 samples. Of these sequences, 78.48% could be classified as AOA sequences by BLAST alignment using the NCBI database, and 4,910–26,206 sequences were obtained per sample (mean 12,398). The read lengths ranged from 376 to 704 bp, with a mean of 513 bp.

**Figure 2:**
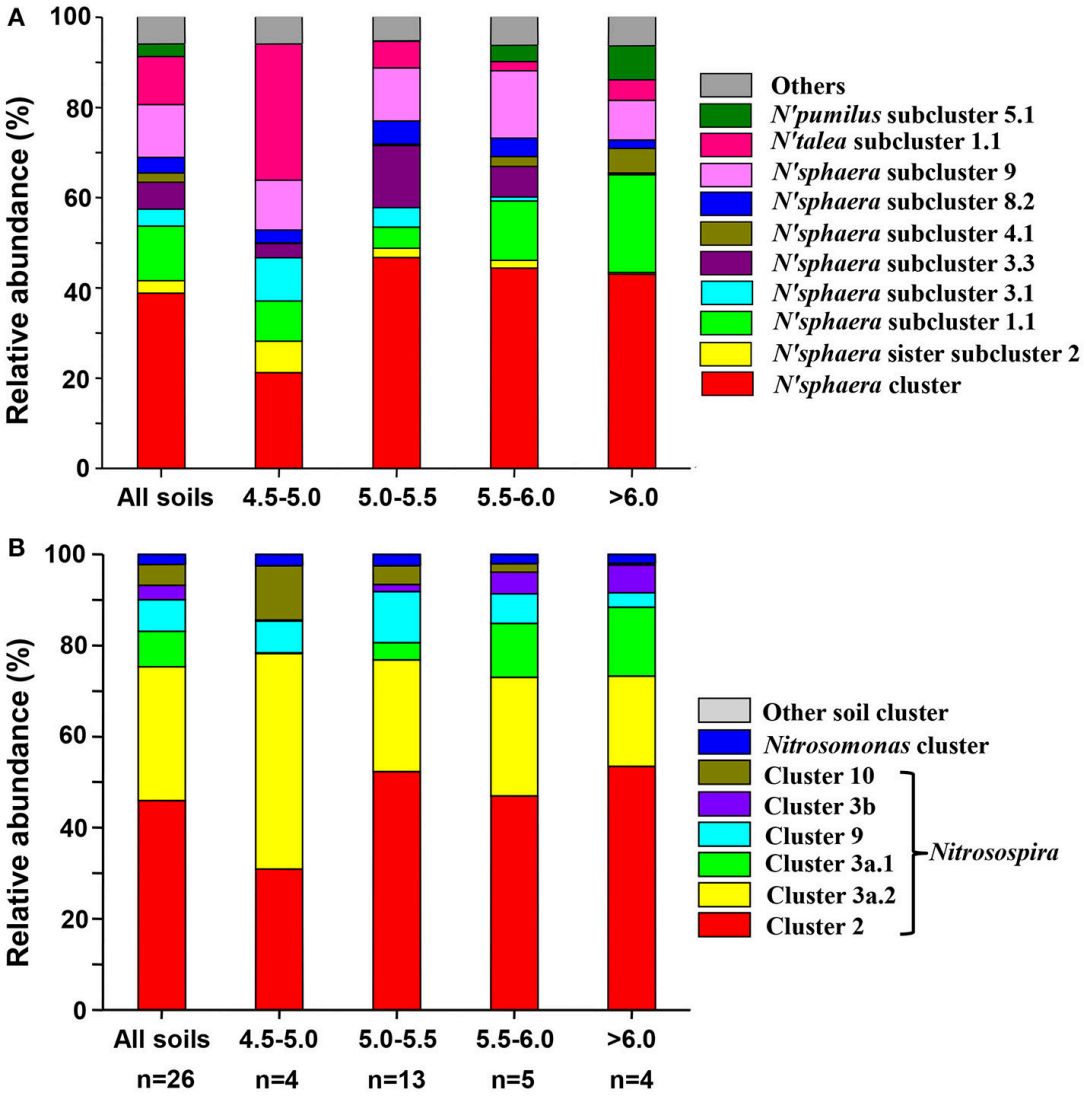
Relative abundances of the different lineages of AOA **(A)** and AOB **(B)** in all soils combined and separately according to pH categories. Relative abundances are based on the proportional frequencies of the DNA sequences that could be classified; Letter n below the column represents the number of samples for each soil pH categories.

The most dominant AOA cluster was *Nitrososphaera*, accounting for an average of 86.22% of total observed sequences (ranged from 39.44 to 98.84%). The second dominant cluster was *Nitrosotalea*, accounting for 8.68% of total sequences (ranged from <0.01 to 50.80%). Sequences belonging to *Nitrososphaera* sister cluster and *Nitrosopumilus* cluster accounted for average 3.12% and 1.98% of the obtained sequences, respectively. According to the taxonomic classification rule proposed by Pester et al. (2012), 15, 2, 2 and 1 subclusters were detected within *Nitrososphaera* cluster, *Nitrososphaera* sister cluster, *Nitrosopumilus* cluster and *Nitrosotalea* cluster, respectively. Detail classification of AOA based on sampling locations was shown in Table S2, and the relative abundances of different lineages of AOA in different pH categories are shown in Figure 2A.

The differentiation of the relative abundance of some AOA lineages was strongly affected by environmental factors such as sampling latitude, soil pH, soil total C and N and soil water content (Table S3). The relative abundance of different AOA lineages correlated weakly with soil C/N ratio, total P, available P and K, ${\text{NH}}_{4}^{+}$-N and ${\text{NO}}_{3}^{-}$-N. In addition, the subclusters within *Nitrososphaera*, such as subclusters 1.1, 2.1, 4.1, 6.1, and 8.1 correlated positively with PNR, while subcluster 3.1 correlated negatively with PNR (Table S3).

Across all 26 soil samples, 237,622 quality sequences of AOB *amoA* were obtained. Of these sequences, 82.89% could be classified as AOB sequences by BLAST alignment using the NCBI database, and 4,155–17,671 sequences were obtained per sample (mean 7,629). The read lengths ranged from 425 to 692 bp, with a mean of 525 bp.

The most abundant AOB sequences across all the samples belonged to the *Nitrosospira* lineage, accounting for 97.71% of the obtained sequences, while the *Nitrosomonas* lineage only occupied 2.28% of the sequences. Within *Nitrosospira* lineage, six clusters (clusters 2, 3a.1, 3a.2, 3b, 9, and 10) were detected across all soils, of which cluster 2 was the most abundant group (48.19%), followed by cluster 3a.2 (27.56%), cluster 9 (8.42%), cluster 3a.1 (6.54%), cluster 10 (4.36%), and cluster 3b (2.65%) (Table S4). Figure 2B showed the relative abundances of different lineages of AOB in different pH categories.

Soil pH value correlated positively with relative abundance of cluster 3a.1 (*r* = 0.795, *p* < 0.0001) and cluster 3b (*r* = 0.428, *p* = 0.003) but negatively correlated with cluster 3a.2 (*r* = −0.475, *p* = 0.001) and cluster 10 (*r* = −0.636, *p* = 0.001) (Table S5). In addition, soil total C, N, and P correlated positively with relative abundance of cluster 2, and negatively with cluster 3a.2 (Table S5). Moreover, the relative abundance of cluster 2 was positively correlated with soil water content and the latitude of sampling location, while the relative abundance of cluster 3a.2 was negatively correlated with the latitude of sampling location (Table S5). Thus, the response of cluster 2 to soil parameters was contrary to that of cluster 3a.2. Furthermore, the relative abundances of cluster 3a.1 was positively related with PNR (*r* = 0.708, *p* < 0.0001), while the relative abundance of cluster 9 (*r* = −0.428, *p* = 0.029) and cluster 10 (*r* = −0.474, *p* = 0.015) negatively correlated with PNR (Table S5).

### Alpha diversity of AOA and AOB

The alpha diversity of both AOA and AOB was measured as OTU richness and PD values at the 85% identity level. There were 63 different AOA phylotypes (OTUs) across all the soils with 21–34 OTUs per sample (a mean of 27). For AOB community, totally 16 different OTUs were obtained from all soils with 7-12 OTUs per sample (a mean of 9). The PD value for AOA and AOB community ranged from 2.75 to 4.33 and from 2.61 to 3.42, respectively.

The OTU richness and PD values of AOA community did not correlate with any soil parameters or latitude of sampling location (Table S6). In contrast, the OTU richness of AOB community correlated positively with soil pH, total P and available K (*r* = 0.425–0.548, *p* < 0.05), and the PD values correlated positively with soil pH, total C, N, and P, available K and soil water content (*r* = 0.466–0.572, *p* < 0.05) (Figure S3 and Table S6). In addition, the OTU richness (*r* = 0.553, *p* = 0.003) and PD values (*r* = 0.601, *p* = 0.001) of AOB community correlated positively with PNR (Table S6). No significant relationship existed between alpha diversity of AOA and PNR (Figure S4 and Table S6).

The neighbor-joining phylogenetic tree of AOA showed that 56 OTUs were grouped into *Nitrosospharea* cluster, 3 to *Nitrosotalea* cluster, 4 to *Nitrospumillus* cluster, but no OTUs were grouped into *Nitrosocaldus* cluster (Figure S5). The tree of AOB showed that 13 and 3 OTUs were grouped into *Nitrosospira* and *Nitrosomonas* cluster, respectively (Figure S6).

### AOA and AOB community structure

**Figure 3:**
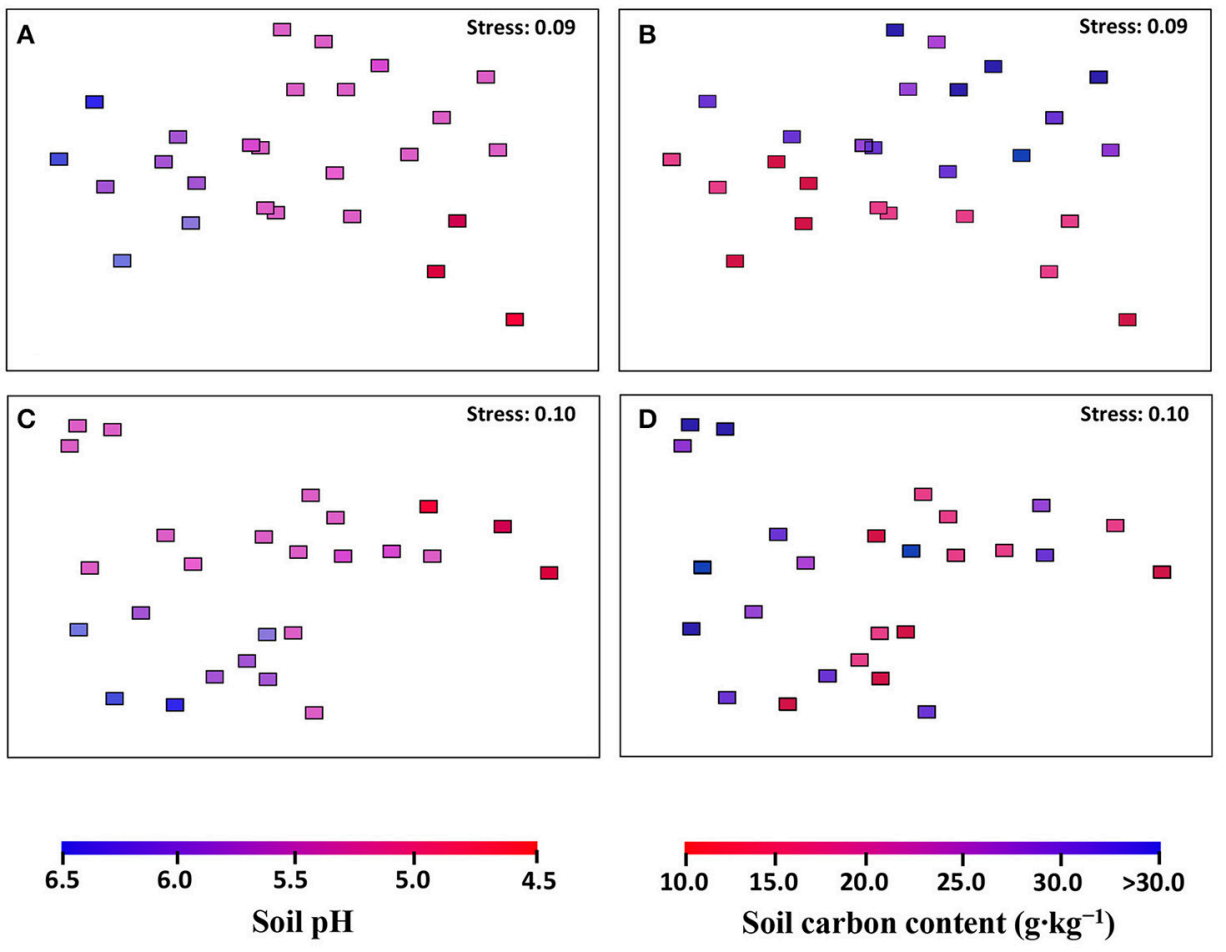
The AOA **(A,B)** and AOB **(C,D)** community structures in black soils as indicated by non-metric multi-dimensional scaling plots of weighted pairwise UniFrac community distances between sites. Sites have been color-coded to gradient of soil pH **(A,C)** and soil total carbon content **(B,D)**.

The community structures of AOA and AOB were illustrated by weighed NMDS plots based on soil pH and soil total C contents (Figure 3). For AOA community, the NMDS1 and NMDS2 axis score was negatively related with soil pH (*r* = −0.685, *p* < 0.001) and positively with soil total C (*r* = 0.226, *p* = 0.006), respectively. While for AOB community, both NMDS1 (*r* = −0.587, *p* = 0.002) and NMDS2 (*r* = −0.607, *p* = 0.001) axis score was negatively related with soil pH (Table 1). In addition, the Mantel test results showed that the AOA community structure was affected significantly by sampling latitude, soil pH, and soil total C and P, while AOB community structure was affected by sampling latitude, soil pH, soil total C, N, and P, and soil water content (Table S7).

**Table 1:** The correlation (*r*) and significance (*p*) values of pairwise regressions between AOA and AOB NMDS scores and soil total C (TC) content, and soil pH value.

**Soil parameter**	**AOA**				**AOB**			
	**NMDS1**		**NMDS2**		**NMDS1**		**NMDS2**	
	**r**	***P***	**r**	***p***	**r**	***p***	**r**	***P***
pH	−**0.685**	<0.001	−0.335	0.094	−**0.587**	0.002	−**0.607**	0.001
TC	−0.333	0.097	**0.462**	0.015	−0.345	0.095	0.178	0.385

*Values in bold indicate significant correlations (p < 0.05)*.

**Figure 4:**
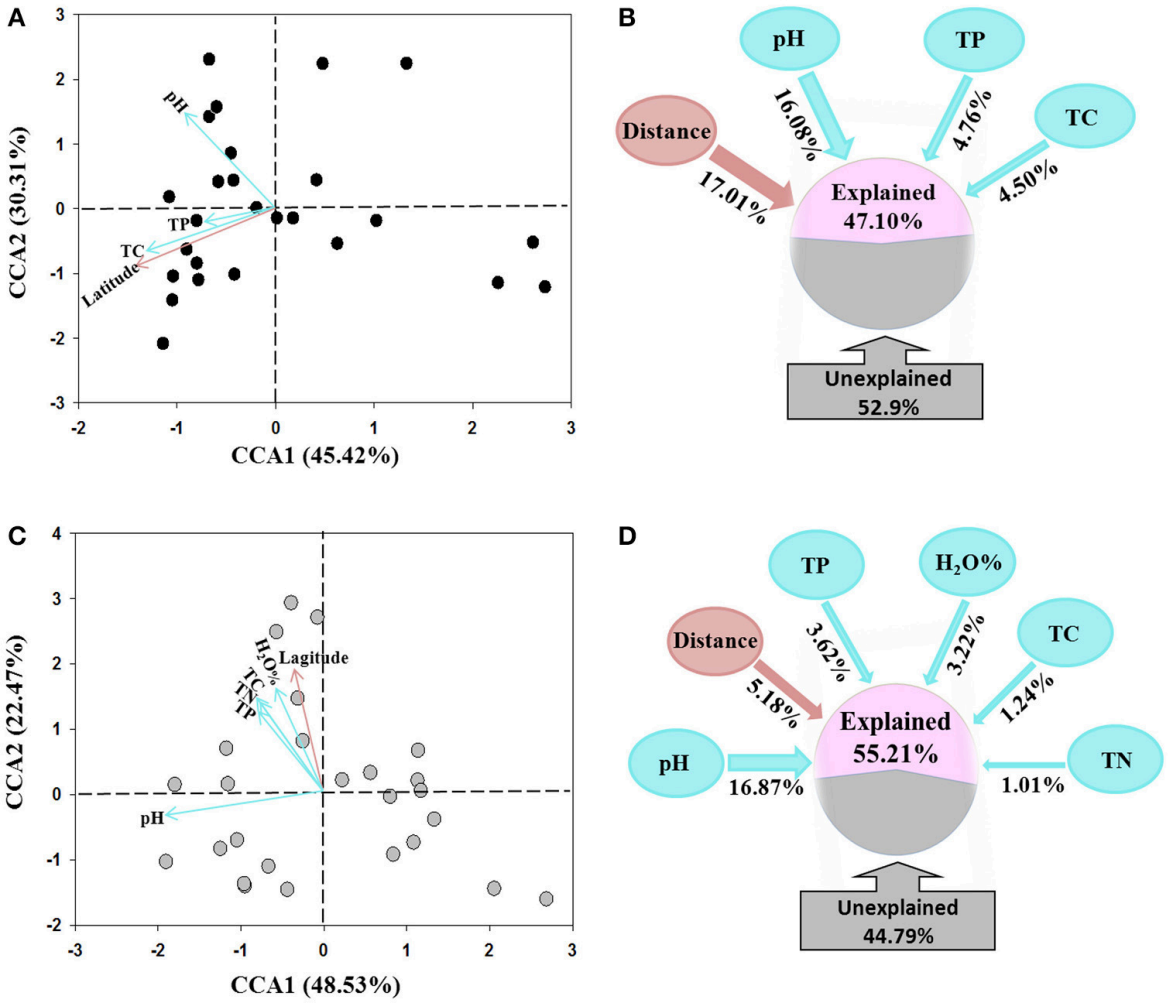
Canonical correspondence analysis (CCA) of environmental factors and pyrosequencing data of AOA **(A)** and AOB **(C)** communities, and the percentages of variance explained by spatial distance and soil variables on the community structure of AOA **(B)** and AOB **(D)** tested using variation partition analysis.

The CCA analysis indicated that the AOA and AOB community structures differed among the sampling locations (Figures 4A,C). Latitude was the dominating factor in shifting community structure of AOA, followed by soil pH, total P and C (Figure 4A). In comparison, the soil pH was determined as the dominating factor influencing AOB community structures, while total P, C, and N, and water content as well as the latitude of sampling locations also contributed to the change of AOB community (Figure 4C).

The VPA analysis was conducted to quantify the relative contributions of the soil parameters and the geographic distance to the AOA and AOB community structures. For AOA community, the geographic distances explained the largest variation of 17.01% the community, the combination of the selected soil parameters explained 25.34% with soil pH was the master variable explaining 16.08% of the community variation, leaving 52.90% of the variation unexplained (Figure 4B). For AOB community, all selected soil parameters explained 25.96% of the community variation with soil pH explaining 16.87% variation and being the dominating factor. The geographic distances only explained 5.18% variation, leaving 44.79% of the variation unexplained (Figure 4D).

**Figure 5:**
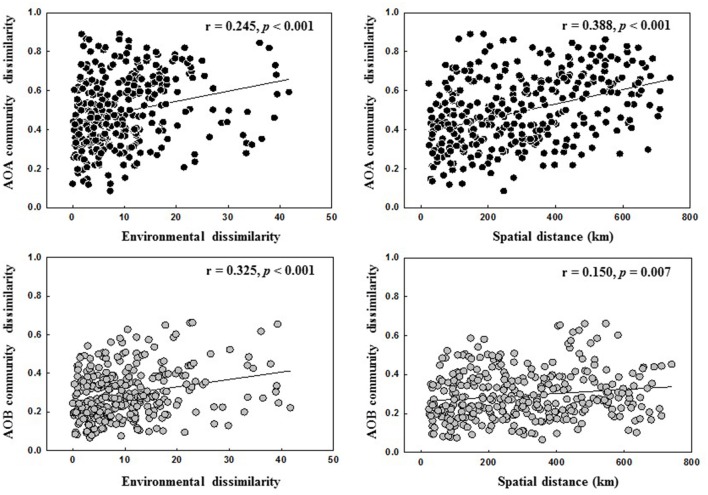
Spearman's correlations between AOA and AOB communities, spatial distance and environmental dissimilarity distance.

The relative contributions of geographic distance (historic factors) and environmental variables (contemporary factors) to AOA and AOB community distribution are shown in Figure 5. Geographic distance had a stronger correlation with AOA community dissimilarities than environmental factors, whereas AOB community was more influenced by environmental factors than geographic distance (Figure 5). This finding was further confirmed by the Partial Mantel tests on the AOA and AOB community composition (Table S8).

## Discussion

Most previous studies have shown the biogeographic distribution of ammonia oxidizers in upland soils (Gubry-Rangin et al., 2011; Pester et al., 2012; Hu et al., 2013, 2014; Yao et al., 2013; Jiang et al., 2014) and paddy soils (Hu et al., 2015). However, the results were not consistent, some studies have indicated that the historic factor of geographic distance explained large proportion variation of AOA and AOB communities (Jiang et al., 2014; Hu et al., 2015), while other studies indicated that the geographic distance explained a lesser extent variation of both communities (Yao et al., 2013). In addition, many studies have indicated that the soil pH is the dominant contemporary factor in shifting both AOA and AOB communities (Hu et al., 2013, 2014, 2015), while Yao et al. (2013) reported that the distribution of AOA and AOB communities in national soil inventory samples of Scotland was driven by multi-factors. To our knowledge, the majority of soil samples in above mentioned studies were collected from different ecosystems, soil types or land use. None of the study was conducted in the same soil type at a large geographic scale. The use of single soil type of black soil in this study removed the influences of ecosystems, soil types or land use which may drive the changes of ammonia oxidizing community. Our findings strongly indicated that ammonia oxidizers were not randomly distributed in the black soil zone, both AOA and AOB displayed the geographic distribution patterns alike to the bacterial and fungal communities in this region (Liu et al., 2014, 2015).

### Larger number of AOA over AOB amoA gene in the black soils

The predominance of AOA over AOB *amoA* gene in the black soils was in line with previous findings in various agricultural soils (Leininger et al., 2006; He et al., 2007; Shen et al., 2012; Jiang et al., 2014) and natural soils (Yao et al., 2013). However, the relationships between abundances of AOA or AOB and soil parameters varied between studies. In the large-scale paddy soils in east China, AOA abundance was significantly affected by total N and sand proportion, and AOB abundance correlated with mean annual precipitation and soil pH (Hu et al., 2015). In the agricultural soils covering uplands and paddy soils with various soil and vegetation types in east China, AOA abundance was significantly correlated with climatic and nutritional factors, while AOB abundance did not correlate with environmental factors (Jiang et al., 2014). Conversely, the abundance of AOA (Gubry-Rangin et al., 2011; Hu et al., 2013, 2014; Yao et al., 2013) and AOB (Hu et al., 2013; Yao et al., 2013) correlated positively with soil pH, with AOB exhibiting a stronger correlation than AOA (Hu et al., 2013; Yao et al., 2013).

In the black soils of this study, the abundance of AOB had a significantly positive relationship with soil pH. In contrast, the abundance of AOA displayed a significant positive correlation with soil total C and N but not soil pH (Figure S2). This finding contradicted with the common knowledge that the majority of AOA isolates to date were from oligotrophic conditions (Hatzenpichler et al., 2008; Walker et al., 2010). Several studies have indicated that AOA are autotrophic microorganisms capable of using carbon dioxide as the sole carbon source (Zhang et al., 2010; Tourna et al., 2011). However, other studies have reported that AOA are heterotrophic or mixtrophic microorganisms in regard to carbon utilization (Hallam et al., 2006; Walker et al., 2010; Zhalnina et al., 2012). Thus, the response of AOA to carbon sources is very complex; increasing carbon inputs can enhance some members but inhibit others (Zhalnina et al., 2012). For an example, Lehtovirta-Morley et al. (2014) found that two closely-related strains of *Nitrosotalea* exhibited differences in physiological features, including growth rate in responses to organic compounds. Our results showed that the relative abundance of several lineages of AOA positively or negatively related with total C and N contents (Table S3), supporting this complex response.

### Distribution of major AOA and AOB lineages in black soils

All known AOA *amoA* sequences have been classified as members of *Nitrosopumilus, Nitrososphaera, Nitrosocaldus, Nitrosotalea*, and *Nitrososphaera* sister clusters (Pester et al., 2012; Jiang et al., 2014). In this study, an unsurprising finding was that the sequence of *Nitrosocaldus* cluster was not detected, since this cluster mainly contains sequences from hot springs (Pester et al., 2012). Sequences belonging to other four clusters were observed in the black soils, and the proportion of sequences distributed into different clusters varied among samples (Table S2). This finding was consistent with that of Pester et al. (2012), who investigated *amoA* sequences of 16 soil samples in Austria, Costa Rica, Greenland and Namibia, and revealed that the largest AOA sequences were affiliated to the *Nitrososphaera* cluster, only small proportion of the sequences were distributed into *Nitrosotalea, Nitrosopumilus* and *Nitrososphaera* sister clusters. Similar findings were also observed in a larger scale survey on the geographic distribution of AOA *amoA* sequences in Chinese soils (Hu et al., 2014, 2015; Jiang et al., 2014).

Many studies have indicated that the distribution of AOA is closely related with soil pH (Nicol et al., 2008; Gubry-Rangin et al., 2011; Hu et al., 2014, 2015; Jiang et al., 2014). Gubry-Rangin et al. (2011) surveyed AOA *amoA* sequences in soils at the global, regional and local scales, and found that geographic distributions of different AOA lineages were determined by specific soil pH ranges. These AOA lineages were classified as acidophilic, acido-neutrophilic and alkalinophilic groups. They also observed that although the relative abundance of lineage B (*Nitrososphaera* cluster) was positively related with soil pH, several subclusters within this lineage were positively or negatively related with soil pH (Gubry-Rangin et al., 2011). In this study, we found that the response of different subclusters within *Nitrososphaera* cluster was not in congruence with soil pH (Table S3), consistent with the results of Gubry-Rangin et al. (2011). Moreover, we further observed the distribution of some lineages of AOA at the subcluster level was also positively or negatively related with the latitude of sampling location, total C and N, and soil moisture (Table S3). This result was in line with previous studies (Yao et al., 2011; Zhalnina et al., 2012; Oton et al., 2016), suggesting that there are multiple soil factors in determining the distribution the lineage of AOA *amoA* gene in the black soils.

*Nitrosospira* and *Nitrosomonas* of the Betaproteobacteria are two dominant genera of terrestrial AOB (Kowalchuk and Stephen, 2001). The *Nitrosospira*-related AOB are abundant in different soils, and they can be grouped into clusters 0, 1, 2, 3a, 3b, 4, 9, 10, 11, and 12 (Avrahami and Conrad, 2003a; Avrahami et al., 2003b). The *Nitrosomonas*-related AOB are grouped into clusters 5, 6a, 6b, 7, and 8 (Freitag and Prosser, 2003), which are frequently found in aquatic environments (Laanbroek and Speksnijder, 2008; Santoro et al., 2008; Hu et al., 2010), sediments (Freitag and Prosser, 2003; Urakawa et al., 2006), and paddy soils (Chen et al., 2010). Hu et al. (2015) surveyed 11 paddy soils across east China, and revealed that approximately 87.5 and 12.5% sequences were classified into the *Nitrosospira* and *Nitrosomonas* lineage, respectively. The relative abundance of *Nitrosospira* in paddy soils was lower while that of *Nitrosomonas* was larger than the corresponding lineage in the black soils of this study (Table S4). In addition, within *Nitrosospira* lineage, the most abundant groups in paddy soils were cluster 11, followed by clusters 3a.1, 3b, 1, 9, 3a.2, 4, and 2 (Hu et al., 2015), while the most abundant groups in the black soils were cluster 2, followed by clusters 3a.2, 9, 3a.1, 10, and 3b (Table S4). Furthermore, the distribution of different lineages in the black soils also differed from the results observed in a wide range of soil and ecosystem types in China, showing that clusters 3a.1 and 3a.2 were the most abundant lineages (Hu et al., 2013). Thus the compositions of AOB communities in the black soils are unique, which are distinctly different from those in paddy soils and other upland soils.

Large-scale investigations showed that the distribution of different AOB lineages in soils was closely related with soil pH (Hu et al., 2013, 2015; Jiang et al., 2014). In this study, we observed the relative abundance of clusters 3a.1 and 10 was positively and negatively related with soil pH, respectively (Table S5). This finding was consistent with the results observed in different upland soils across China (Hu et al., 2013). However, we also observed the clusters 3b and 3a.2 was positively and negatively correlated with soil pH, respectively (Table S5), which was not observed by Hu et al. (2013). Furthermore, Hu et al. (2013) found that cluster 2 was negatively related with soil pH, while this cluster was not related with soil pH in the black soils (Table S5). The inconsistent relations of different AOB lineages to soil pH might be related to the different pH ranges between this study and previous studies (Hu et al., 2013, 2015; Jiang et al., 2014). The range of pH values in this study was narrower than the previous large-scale investigations (Hu et al., 2013, 2015; Jiang et al., 2014), which may lead to the impact of pH on the distribution of AOB lineages in the black soils being masked by other environmental factors (Jiang et al., 2014). Indeed, we observed other parameters such as the sampling latitude, total C, N and P, and water content were closely related with the distribution of certain AOB lineages in the black soils (Table S5).

### Comparison of geographic distribution AOA and AOB communities in the black soil zone

Many studies have reported that AOA and AOB community structures in soils are affected by multiple environmental conditions (Shen et al., 2012; Zhalnina et al., 2012; Yao et al., 2013). Previous large-scale studies showed that the contribution of historical factor of geographic distance and contemporary factor of soil parameters to the overall AOA and AOB community structures varied with studies. For example, both AOA and AOB communities in paddy fields exhibited the classical distance-decay patterns and geographic distance contributed equally to the variation of both communities (Hu et al., 2015). In contrast, Jiang et al. (2014) revealed that geographic distance explained a greater variation of AOB community than AOA community across different soil types of agricultural lands (AOB: 20.3% vs. AOA: 12.4%). Our present study also showed that the community structures of both AOA and AOB were determined by multiple environmental factors in the black soils (Figure 4). Among them, the historical factor of geographic distance explained a larger variation of AOA community than AOB community (17.01 vs. 5.18%), suggesting that the biogeographic distribution of AOA community in the black soil zone was more pronounced than AOB community, and this finding was further corroborated by the linear regression analysis of relationship between geographic distance and community dissimilarity of AOA and AOB with the *r*-value being greater for AOA (*r* = 0.388) than for AOB community (*r* = 0.150) (Figure 5).

Soil pH has been commonly detected as the major soil factor in determining soil bacterial community in various ecosystems (Fierer and Jackson, 2006; Lauber et al., 2009; Chu et al., 2010; Rousk et al., 2010; Griffiths et al., 2011). Our previous studies also revealed that soil pH was the most important edaphic factor driving the distribution of total bacterial and acidobacterial communities in the black soil zone of northeast China (Liu et al., 2014, 2016). In the present study, soil pH was also determined as the dominating soil factor affecting the distribution of both AOA and AOB communities in the black soils (Figure 4). This result was consistent with the findings of previous studies (Gubry-Rangin et al., 2011; Hu et al., 2013, 2014, 2015), suggesting that soil pH forms a niche to segregate or filter the ammonia-oxidizing microorganisms in soils (Hu et al., 2014). The underlying mechanisms of soil pH are complex, the direct or indirect pH-associated influences such as impact of bioavailability of multiple soil nutrients might result in the changes of AOA and AOB communities (Hu et al., 2015).

### Link PNR to AOA and AOB community in the black soils

Although the abundances of AOA *amoA* gene are larger than those of AOB in most agricultural soils (Shen et al., 2012; Hu et al., 2014, 2015; Ouyang et al., 2016), the rate of ammonia oxidation may or may not be directly related to the AOA and AOB abundances (Nicol et al., 2008; Myrold et al., 2014). It is well known that *amoA* abundance and community composition of AOA are more positively correlated with PNR than those of AOB in acid soils (He et al., 2007; Shen et al., 2008; Yao et al., 2011; Zhang et al., 2012), while the AOB community has been demonstrated to be more important than AOA community for ammonia oxidization in neutral and alkaline soils (Di et al., 2009; Jia and Conrad, 2009; Shen et al., 2011; Xia et al., 2011). Recently, a large-scale investigation in paddy soils showed that the abundances of AOA and AOB were significantly and positively correlated with PNR (Hu et al., 2015) with the correlation being stronger with AOB (*r* = 0.724) than with AOA (*r* = 0.406). In addition, Zhou et al. (2015) surveyed 28 sediments of inland waters across China and found that only abundance of AOB was significantly and positively correlated with PNR (*p* < 0.0001). In the present study, although the soil was slightly acid, we observed that both AOA and AOB abundances were positively correlated with PNR, and the correlation was stronger with AOB (*r* = 0.666, *p* < 0.001) than with AOA (*r* = 0.392, *p* = 0.048) (Table S6). In addition, we also revealed that the alpha diversity of AOB but not AOA positively correlated with PNR (Figure S4 and Table S6). These findings suggested that it was AOB rather than AOA contributed largely to ammonia oxidization in the black soils when high ammonium available concentration in soil as previous studies described (Taylor et al., 2010; Carey et al., 2016; Ouyang et al., 2017). However, since the abundance of AOA was larger than that of AOB, and the correlation between PNR and relative abundances of different lineages of AOA and AOB varied greatly (Tables S3, S5), thus more detailed experiments such as using different inhibitors (Amberger, 1989; Taylor et al., 2013; Daebeler et al., 2015) or DNA-SIP method (Jia and Conrad, 2009) should be conducted in the future to assess the relative contribution of AOA and AOB to ammonia oxidization in the black soils.

In conclusion, this study revealed that the functional microorganisms of AOA and AOB were not stochastically distributed in the black soils, but presented a distinct biogeographic distribution pattern. The geographic distance explained more variation of AOA than AOB community, which suggested that the biogeographic distribution of AOA was more noticeable than AOB community. The copy number of AOA *amoA* gene was larger than that of AOB, consistent with well-documented findings in other agricultural soils. The abundance of AOA *amoA* gene positively correlated with soil total C content while the abundance of AOB *amoA* gene positively correlated with soil pH. Among the tested soil parameters, soil pH was the predominating factor in shaping both AOA and AOB community structures. In addition, several soil parameters such as pH, total C and N, and soil water contents were closely associated to the distribution of different lineages of ammonia oxidizers. Finally, PNR showed a higher correlation with diversity and abundance of AOB than AOA community, which inferred that it was AOB rather than AOA might make a major contribution to ammonia oxidation in the black soils. Further research using more precise and more detailed designs should confirm this speculation.

## Author contributions

Conception of the study: GW, JL, and ZY. Designed the experiments: GW, JJ, and XL. Performed the experiments: QY and YYS. Interpretation of the results: YS and HC. Wrote the manuscript: JL, GW, CT, and AF.

### Conflict of interest statement

The authors declare that the research was conducted in the absence of any commercial or financial relationships that could be construed as a potential conflict of interest.

## Supplementary material

Figure S1 Map of sampling locations in this study.

Figure S2 The linear relationships between abundance of AOA and AOB and soil pH, soil total carbon and soil total nitrogen.

Figure S3 The linear relationships between AOA and AOB OTU phylotype richness or phylogenetic diversity and soil pH, and soil total carbon content. The communities were randomly sampled at the 4900 and 4100 sequences for AOA and AOB *amoA* gene respectively for calculation of alpha diversity at 85% sequence similarity.

Figure S4 The linear relationships between AOA and AOB OTU phylotype richness or phylogenetic diversity and potential nitrification rates. The communities were randomly sampled at the 4900 and 4100 sequences for AOA and AOB *amoA* gene respectively for calculation of alpha diversity at 85% sequence similarity.

Figure S5 Neighbor-joining phylogenetic tree showing the phylogenetic affiliations of the representative sequences of AOA *amoA* gene retrieved from the black soils. The scale bar represents 5% nucleic acid sequence divergence, and bootstrap values (>50%) are indicated at branch points.

Figure S6 Neighbor-joining phylogenetic tree showing the phylogenetic affiliations of the representative sequences of AOB *amoA* gene retrieved from the black soils. The scale bar represents 5% nucleic acid sequence divergence, and bootstrap values (>50%) are indicated at branch points.

Table S1 Locations, soil physical and chemical properties, and the abundances of AOA and AOB *amoA* gene, as well as potential nitrification rate in 26 black soils for this study.

Table S2 Relative abundances (%) of AOA subgroups across all soil samples.

Table S3 The relationships between soil physicochemical properties and the relative abundance of AOA subclusters in black soils using Spearman's correlation. Correlations with significant values (^*^*p* < 0.05; ^**^*p* < 0.01) are shown in bold number.

Table S4 Relative abundances (%) of AOB subgroups across all soil samples.

Table S5 The relationships between soil physicochemical properties and the relative abundance of AOB subclusters in black soils using Spearman's correlations. Correlations with significant values (^*^*p* < 0.05; ^**^*p* < 0.01) are shown in bold number.

Table S6 The relationships between the AOA and AOB abundance, OTU phylotype richness and phylogenetic diversity and soil physicochemical properties and potential nitrification rates. The communities were randomly sampled at the 4900 and 4100 sequences for AOA and AOB *amoA* genes respectively, for calculation of alpha diversity at 85% sequence similarity. Correlations with significant values (^*^*p* < 0.05; ^**^*p* < 0.01) are shown in bold number.

Table S7 The correlations (r) and significance (*p*) determined by Mantel tests between community structure and soil physicochemical properties and potential nitrification rates. Correlations with significant values are shown in bold number.

Table S8 Mantel and Partial Mantel tests were conducted to compare the relative impact of spatial distance and environmental dissimilarity distance on the AOA and AOB communities.
