# Supplementary figures and images for: Ammonia-Oxidizing Archaea Show More Distinct Biogeographic Distribution Patterns than Ammonia-Oxidizing Bacteria across the Black Soil Zone of Northeast China
Source: Front Microbiol. 2018 Feb 9;9:171. doi: 10.3389/fmicb.2018.00171 (PMC5819564; doi:10.3389/fmicb.2018.00171)

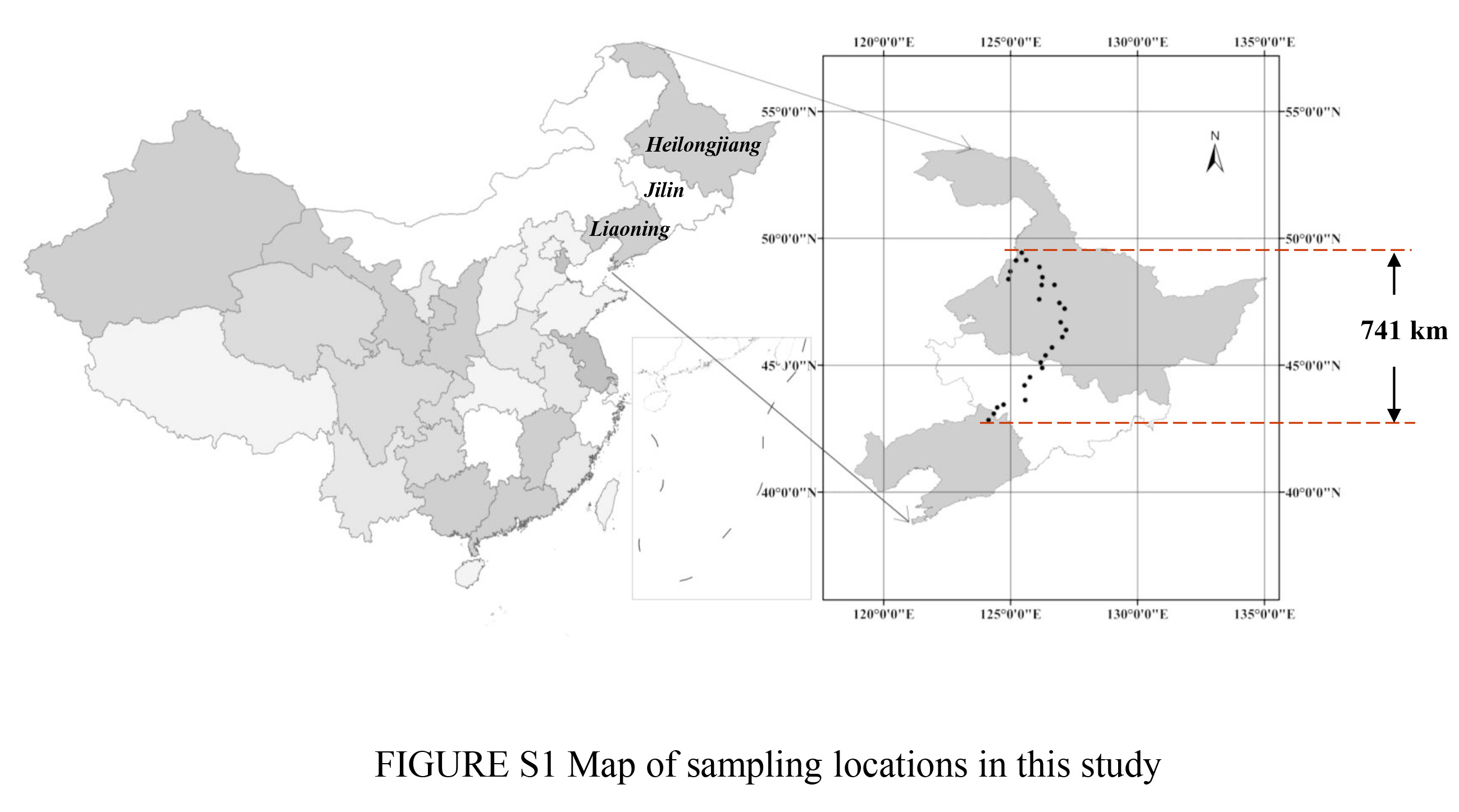

Supplement: Figure S1 — Map of sampling locations in this study. [file Image1.JPEG]

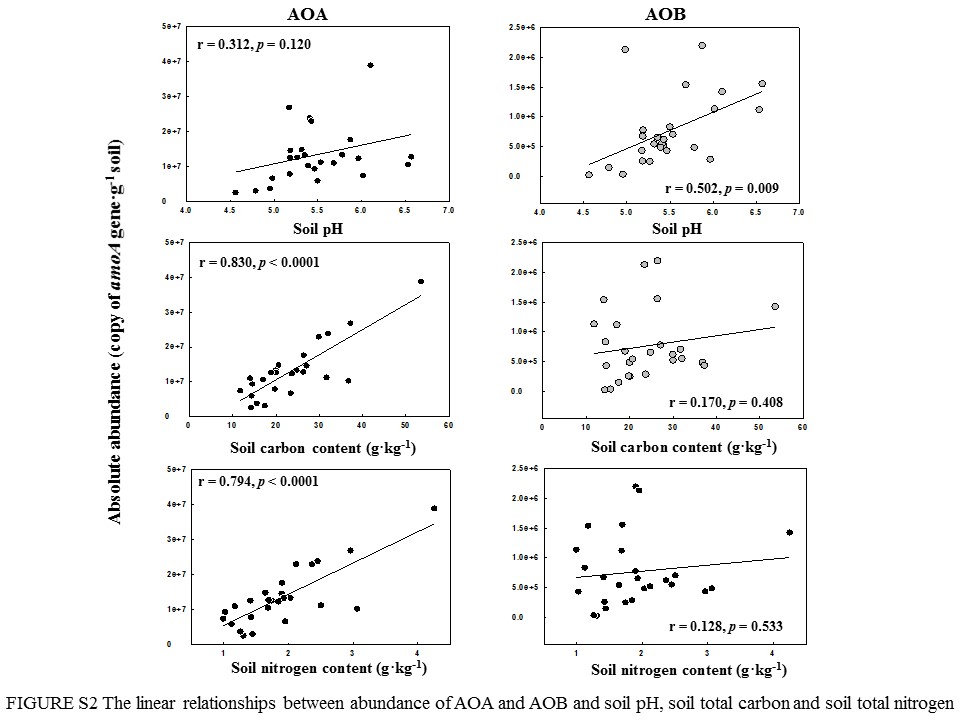

Supplement: Figure S2 — The linear relationships between abundance of AOA and AOB and soil pH, soil total carbon and soil total nitrogen. [file Image2.JPEG]

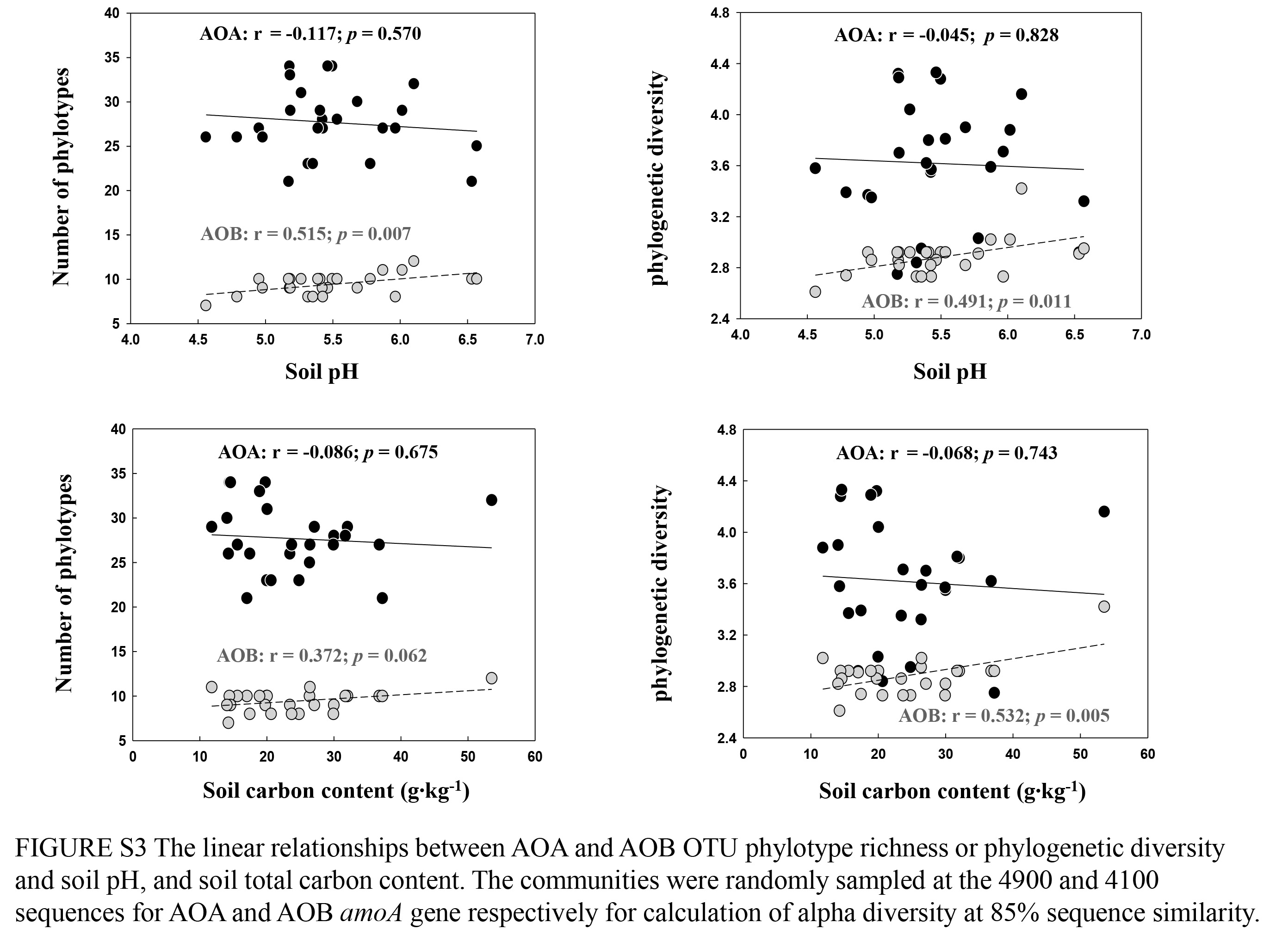

Supplement: Figure S3 — The linear relationships between AOA and AOB OTU phylotype richness or phylogenetic diversity and soil pH, and soil total carbon content. The communities were randomly sampled at the 4900 and 4100 sequences for AOA and AOB amoA gene respectively for calculation of alpha diversity at 85% sequence similarity. [file Image3.JPEG]

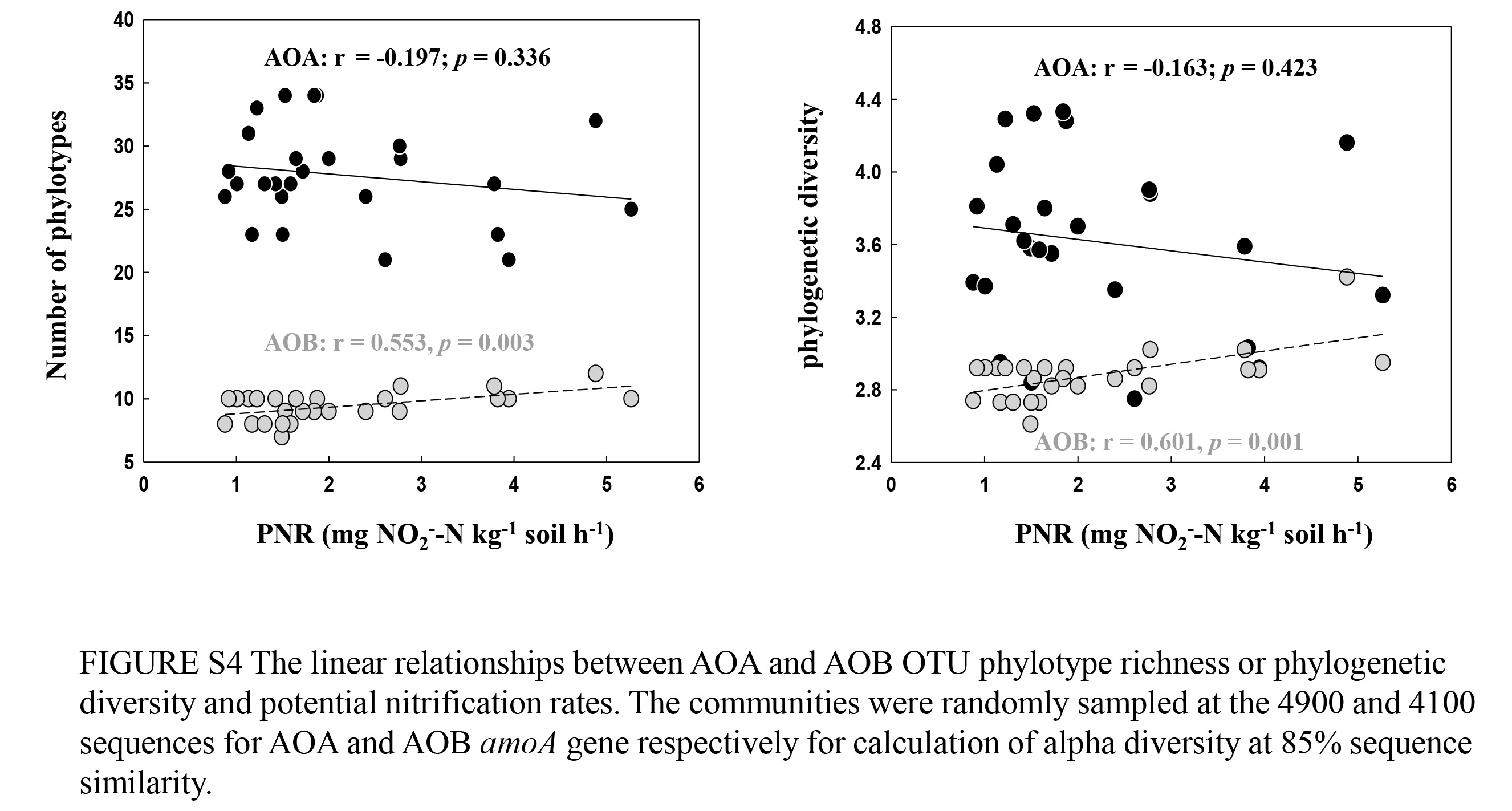

Supplement: Figure S4 — The linear relationships between AOA and AOB OTU phylotype richness or phylogenetic diversity and potential nitrification rates. The communities were randomly sampled at the 4900 and 4100 sequences for AOA and AOB amoA gene respectively for calculation of alpha diversity at 85% sequence similarity. [file Image4.JPEG]

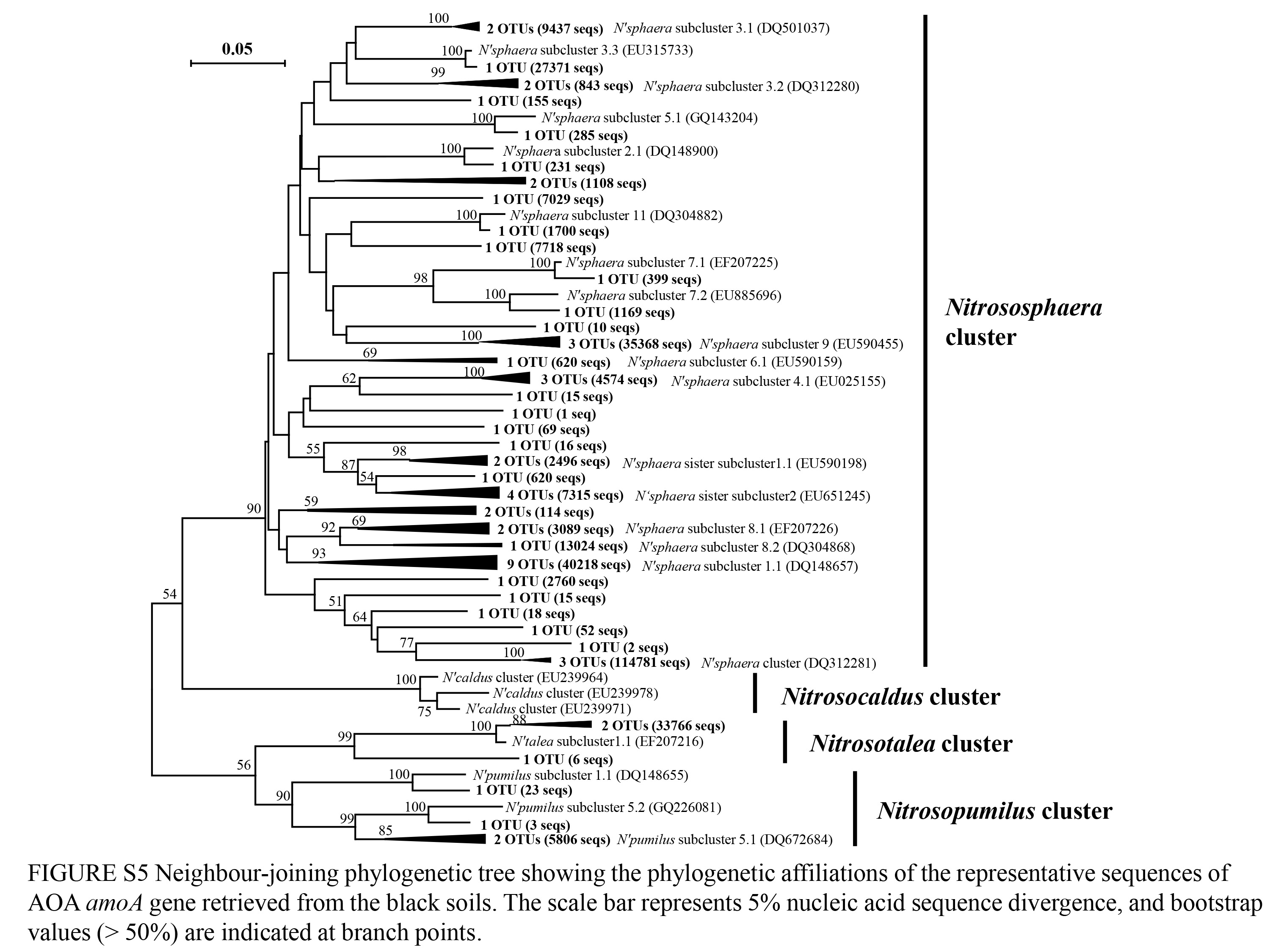

Supplement: Figure S5 — Neighbor-joining phylogenetic tree showing the phylogenetic affiliations of the representative sequences of AOA amoA gene retrieved from the black soils. The scale bar represents 5% nucleic acid sequence divergence, and bootstrap values (>50%) are indicated at branch points. [file Image5.JPEG]

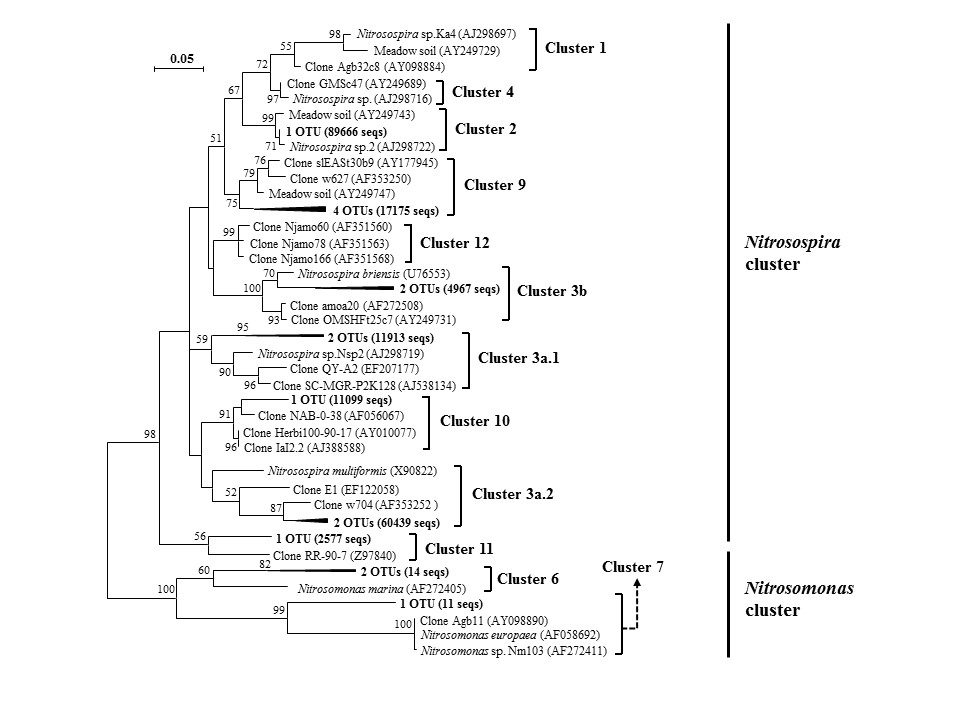

Supplement: Figure S6 — Neighbor-joining phylogenetic tree showing the phylogenetic affiliations of the representative sequences of AOB amoA gene retrieved from the black soils. The scale bar represents 5% nucleic acid sequence divergence, and bootstrap values (>50%) are indicated at branch points. [file Image6.JPEG]
