# Supplementary material for: Ammonia-Oxidizing Archaea Show More Distinct Biogeographic Distribution Patterns than Ammonia-Oxidizing Bacteria across the Black Soil Zone of Northeast China
Source: Front Microbiol. 2018 Feb 9;9:171. doi: 10.3389/fmicb.2018.00171 (PMC5819564; doi:10.3389/fmicb.2018.00171)
Supplement: Table S1 — Locations, soil physical and chemical properties, and the abundances of AOA and AOB amoA gene, as well as potential nitrification rate in 26 black soils for this study. [file Table1.DOC]

**TABLE S1** Locations, soil physical and chemical properties, and the abundances of AOA and AOB in 26 black soils for this study

| Sample | location | Latitude/Longitude | Crop | pH  (H_2_O) | TC^a^  (g kg^−1^) | TN  (g kg^−1^) | H_2_O  (%) | TP  (g kg^−1^) | AK  (mg kg^−1^) | AP  (mg kg^−1^) | NH_4_^+^-N  (mg kg^−1^) | NO_3_^-^-N  (mg kg^−1^) | AOA (copy number  10^6^ g-^1^ soil) | AOB (copy number  10^5^ g-^1^ soil) | AOA/AOB  abundance | PNR (mg NO_2_^-^-N kg^-1^ soil h^-1^) |
| --- | --- | --- | --- | --- | --- | --- | --- | --- | --- | --- | --- | --- | --- | --- | --- | --- |
| CT1 | Cangtu 1, Liaoning | 42°50′N/124°07′E | Maize | 5.68 | 14.04 | 1.17 | 22 | 0.90 | 106.9 | 52.00 | 10.24 | 12.16 | 10.5 | 11.2 | 9.35 | 2.76 |
| CT2 | Cangtu 2, Liaoning | 43°05′N/124°20′E | Maize | 5.46 | 14.58 | 1.02 | 21 | 0.63 | 97.2 | 18.50 | 8.87 | 6.71 | 13.3 | 4.89 | 27.22 | 1.84 |
| LS | Lishu, Jilin | 43°20′N/124°28′E | Maize | 6.02 | 11.77 | 0.99 | 25 | 0.65 | 90.6 | 17.00 | 8.98 | 11.66 | 12.5 | 2.56 | 49.09 | 2.77 |
| GZL | Gongzhuling, Jilin | 43°26′N/124°43′E | Maize | 5.50 | 14.40 | 1.12 | 23 | 0.90 | 110.5 | 31.50 | 9.70 | 8.67 | 2.51 | 0.29 | 85.73 | 1.87 |
| CC | Changchun, Jilin | 43°37′N/125°34′E | Maize | 4.95 | 15.59 | 1.26 | 21 | 1.45 | 114.0 | 48.00 | 9.25 | 8.11 | 3.02 | 1.53 | 19.73 | 1.01 |
| DH1 | Dehui 1, Jilin | 44°12′N/125°33′E | Maize | 4.79 | 17.45 | 1.44 | 22 | 0.74 | 110.5 | 40.50 | 9.74 | 13.07 | 3.69 | 0.41 | 91.04 | 0.88 |
| DH2 | Dehui 2, Jilin | 44°31′N/125°45′E | Maize | 4.56 | 14.26 | 1.30 | 22 | 0.80 | 81.5 | 28.00 | 10.77 | 28.47 | 5.88 | 8.34 | 7.05 | 1.49 |
| YS | Yushu, Jilin | 44°53′N/126°14′E | Maize | 5.27 | 20.03 | 1.74 | 22 | 0.81 | 127.8 | 18.50 | 8.75 | 9.01 | 7.36 | 11.4 | 6.48 | 1.13 |
| FY | Fuyu, Jilin | 45°06′N/126°11′E | Maize | 5.78 | 19.97 | 2.03 | 18 | 0.86 | 106.9 | 16.00 | 9.68 | 7.48 | 9.29 | 4.35 | 21.36 | 3.82 |
| SC | Shuangcheng, Heilongjiang | 45°23′N/126°22′E | Maize | 6.53 | 17.02 | 1.68 | 23 | 0.98 | 106.4 | 29.50 | 9.33 | 8.55 | 11.0 | 15.4 | 7.12 | 3.94 |
| HRB | Harbin, Heilongjiang | 45°41′N/126°38′E | Soybean | 6.57 | 26.36 | 1.69 | 23 | 1.40 | 159.9 | 66.50 | 9.61 | 7.53 | 12.7 | 15.6 | 8.19 | 5.27 |
| HL1 | Hulan, Heilongjiang | 46°06′N/127°02′E | Maize | 5.18 | 19.76 | 1.42 | 22 | 0.75 | 96.7 | 17.00 | 10.03 | 9.38 | 7.82 | 2.63 | 29.74 | 1.53 |
| BY | Bayan, Heilongjiang | 46°23′N/127°11′E | Maize | 5.87 | 26.41 | 1.90 | 25 | 1.14 | 169.5 | 50.00 | 11.89 | 18.43 | 17.6 | 21.9 | 8.02 | 3.79 |
| SH | Suihua, Heilongjiang | 46°41′N/126°58′E | Maize | 5.18 | 18.91 | 1.41 | 24 | 0.83 | 114.5 | 28.00 | 10.97 | 10.18 | 12.5 | 6.77 | 18.50 | 1.22 |
| SL | Suiling, Heilongjiang | 47°13′N/127°07′E | Maize | 5.19 | 27.07 | 1.90 | 30 | 0.68 | 97.7 | 38.00 | 13.33 | 9.60 | 14.5 | 7.82 | 18.58 | 2.00 |
| HL | Hailun, Heilongjiang | 47°27′N/126°55′E | Maize | 5.42 | 29.97 | 2.12 | 27 | 1.15 | 106.9 | 25.00 | 10.84 | 6.45 | 23.0 | 5.24 | 43.82 | 1.72 |
| BQ | Baiquan, Heilongjiang | 47°35′N/126°07′E | Maize | 4.98 | 23.41 | 1.95 | 26 | 0.85 | 130.3 | 42.50 | 34.54 | 70.97 | 6.63 | 21.3 | 3.11 | 2.40 |
| KD | Kedong, Heilongjiang | 48°09′N/126°13′E | Soybean | 5.41 | 32.03 | 2.45 | 24 | 1.08 | 102.3 | 31.50 | 9.73 | 20.50 | 23.8 | 5.54 | 42.97 | 1.64 |
| BA | Beian, Heilongjiang | 48°09′N/126°43′E | Soybean | 6.10 | 53.53 | 4.25 | 40 | 1.50 | 151.7 | 36.00 | 13.01 | 16.85 | 38.8 | 14.2 | 27.27 | 4.88 |
| WC1 | Wudalianchi 1, Heilongjiang | 48°28′N/126°15′E | Soybean | 5.43 | 29.92 | 2.36 | 28 | 1.14 | 96.7 | 27.00 | 10.89 | 12.57 | 22.9 | 6.25 | 36.64 | 1.59 |
| WC2 | Wudalianchi 2, Heilongjiang | 48°52′N/126°08′E | Soybean | 5.39 | 36.76 | 3.06 | 35 | 1.33 | 125.7 | 22.00 | 11.12 | 14.06 | 10.2 | 4.91 | 20.73 | 1.42 |
| NH1 | Nehe 1, Heilongjiang | 48°41′N/124°59′E | Maize | 5.35 | 24.78 | 1.93 | 28 | 0.93 | 78.9 | 27.50 | 10.35 | 9.94 | 11.2 | 7.09 | 15.76 | 1.17 |
| NH2 | Nehe 2, Heilongjiang | 48°23′N/124°55′E | Soybean | 5.97 | 23.68 | 1.84 | 25 | 0.87 | 115.1 | 25.00 | 9.80 | 10.60 | 26.8 | 4.38 | 61.24 | 1.31 |
| NJ1 | Nenjiang 1, Heilongjiang | 49°08′N/125°37′E | Maize | 5.53 | 31.71 | 2.50 | 28 | 1.30 | 103.9 | 42.50 | 9.91 | 13.11 | 14.8 | 5.46 | 27.03 | 0.92 |
| NJ2 | Nenjiang 2, Heilongjiang | 49°26′N/125°26′E | Wheat | 5.17 | 37.23 | 2.96 | 33 | 1.23 | 132.4 | 24.50 | 11.74 | 11.00 | 13.3 | 6.58 | 20.15 | 2.61 |
| NJ3 | Nenjiang 3, Heilongjiang | 49°07′N/125°13′E | Soybean | 5.32 | 20.63 | 1.64 | 26 | 0.96 | 122.2 | 42.00 | 10.00 | 9.46 | 12.3 | 2.91 | 42.25 | 1.50 |

^a^ TC: total carbon; TN: total nitrogen; TP: total phosphorus; AK: available potassium content; AP: available phosphorus; H_2_O%: soil water content; NH_4_^+^: ammonium, NO_3_^-^: nitrate; PNR: potential nitrification rate.
