# Supplementary material for: Ammonia-Oxidizing Archaea Show More Distinct Biogeographic Distribution Patterns than Ammonia-Oxidizing Bacteria across the Black Soil Zone of Northeast China
Source: Front Microbiol. 2018 Feb 9;9:171. doi: 10.3389/fmicb.2018.00171 (PMC5819564; doi:10.3389/fmicb.2018.00171)
Supplement: Table S3 — The relationships between soil physicochemical properties and the relative abundance of AOA subclusters in black soils using Spearman's correlation. Correlations with significant values (*p < 0.05; **p < 0.01) are shown in bold number. [file Table3.DOCX]

| AOA | Latitude | pH | TC | TN | C/N | H_2_O% | TP | AK | AP | NH_4_^+^-N | NO_3_^-^-N | PNR |
| --- | --- | --- | --- | --- | --- | --- | --- | --- | --- | --- | --- | --- |
| *N'pumilus* cluster | | | | | | | | | | | | |
| *N'pumilus* cluster | -0.013 | **0.394*** | -0.107 | -0.193 | 0.360 | -0.187 | 0.049 | **0.467*** | **0.506**** | 0.063 | 0.071 | 0.357 |
| Subcluster 1.1 | -0.386 | -0.175 | -0.324 | -0.322 | -0.073 | -0.154 | -0.235 | -0.235 | -0.022 | -0.066 | 0.012 | -0.081 |
| Subcluster 5.1 | **-0.438*** | 0.244 | -0.349 | -0.331 | -0.163 | -0.093 | -0.289 | -0.223 | -0.060 | -0.110 | -0.054 | 0.138 |
| *N'sphaera* sister cluster | | | | | | | | | | | | |
| Subcluster 1.1 | **-0.555**** | 0.165 | -0.313 | -0.303 | -0.117 | -0.245 | 0.119 | 0.050 | **0.403*** | -0.139 | -0.111 | 0.167 |
| Subcluster 2 | -0.005 | **-0.647**** | -0.113 | -0.075 | -0.192 | -0.033 | -0.185 | -0.222 | -0.008 | 0.257 | **0.426*** | **-0.433*** |
| *N'sphaera* cluster | | | | | | | | | | | | |
| N'sphaera cluster | **0.495*** | 0.260 | **0.457*** | **0.464*** | 0.015 | 0.254 | 0.072 | 0.191 | -0.276 | 0.066 | -0.028 | 0.267 |
| Subcluster 1.1 | -0.340 | **0.635**** | -0.253 | -0.278 | 0.098 | **-0.420*** | 0.208 | **0.404*** | **0.488*** | -0.109 | -0.110 | **0.649**** |
| Subcluster 2.1 | -0.105 | **0.693**** | 0.198 | 0.155 | 0.198 | 0.010 | 0.277 | 0.291 | 0.332 | -0.151 | -0.189 | **0.734**** |
| Subcluster 3.1 | **-0.478*** | **-0.419*** | **-0.432*** | **-0.448*** | 0.039 | **-0.402*** | -0.150 | -0.199 | -0.045 | -0.180 | -0.156 | **-0.418*** |
| Subcluster 3.2 | -0.184 | -0.384 | -0.092 | -0.062 | -0.122 | -0.060 | 0.188 | -0.004 | 0.208 | -0.122 | -0.082 | -0.379 |
| Subcluster 3.3 | **0.553**** | -0.218 | **0.466*** | **0.452*** | 0.113 | **0.550**** | 0.300 | -0.200 | -0.171 | -0.061 | -0.124 | -0.373 |
| Subcluster 4.1 | 0.025 | **0.404*** | **0.504**** | **0.562**** | -0.201 | **0.447*** | 0.291 | 0.289 | 0.001 | 0.035 | 0.026 | **0.603**** |
| Subcluster 5.1 | 0.144 | -0.273 | 0.169 | 0.234 | -0.227 | 0.306 | 0.197 | -0.080 | -0.127 | -0.042 | 0.103 | -0.253 |
| Subcluster 6.1 | **-0.506**** | **0.563**** | -0.294 | -0.310 | 0.05 | -0.299 | -0.097 | 0.069 | 0.270 | -0.192 | -0.179 | **0.539**** |
| Subcluster 7 | **0.409*** | -0.145 | **0.426*** | **0.441*** | 0.034 | **0.545**** | 0.314 | -0.017 | -0.141 | -0.048 | -0.080 | -0.280 |
| Subcluster 7.1 | 0.349 | -0.022 | **0.551**** | **0.610**** | -0.18 | **0.614**** | **0.458*** | 0.099 | -0.043 | -0.006 | 0.111 | -0.014 |
| Subcluster 7.2 | 0.335 | -0.045 | 0.337 | 0.381 | -0.088 | **0.444*** | 0.365 | 0.041 | -0.091 | -0.038 | -0.020 | -0.227 |
| Subcluster 8.1 | **-0.434*** | **0.662**** | -0.196 | -0.146 | -0.19 | -0.317 | -0.003 | 0.157 | 0.233 | -0.128 | -0.155 | **0.730**** |
| Subcluster 8.2 | 0.258 | -0.115 | 0.053 | 0.023 | 0.141 | 0.138 | -0.107 | -0.071 | -0.155 | 0.172 | 0.081 | -0.362 |
| Subcluster 9 | 0.100 | 0.111 | -0.219 | -0.247 | 0.133 | -0.233 | -0.197 | 0.231 | 0.231 | **0.420*** | 0.387 | 0.012 |
| Subcluster 11 | **0.480*** | -0.378 | **0.538**** | **0.536**** | 0.009 | **0.606**** | 0.288 | -0.170 | -0.144 | 0.011 | 0.072 | -0.187 |
| *N'talea* cluster | | | | | | | | | | | | |
| Subcluster 1.1 | -0.013 | **0.394*** | -0.107 | -0.193 | 0.36 | -0.187 | 0.049 | **0.467*** | **0.506**** | 0.063 | 0.071 | 0.357 |

**TABLE S3** The relationships between soil physicochemical properties and the relative abundance of AOA subclusters in black soils using Spearman’s correlation. Correlations with significant values (**^*^**, *p* < 0.05; **^**^**, *p* < 0.01) are shown in bold number.
