# Supplementary material for: Ammonia-Oxidizing Archaea Show More Distinct Biogeographic Distribution Patterns than Ammonia-Oxidizing Bacteria across the Black Soil Zone of Northeast China
Source: Front Microbiol. 2018 Feb 9;9:171. doi: 10.3389/fmicb.2018.00171 (PMC5819564; doi:10.3389/fmicb.2018.00171)
Supplement: Table S5 — The relationships between soil physicochemical properties and the relative abundance of AOB subclusters in black soils using Spearman's correlations. Correlations with significant values (*p < 0.05; **p < 0.01) are shown in bold number. [file Table5.DOCX]

**TABLE S6** The relationships between the AOA and AOB abundance, OTU phylotype richness and phylogenetic diversity and soil physicochemical properties and potential nitrification rates. The communities were randomly sampled at the 4900 and 4100 sequences for AOA and AOB *amoA* genes respectively, for calculation of alpha diversity at 85% sequence similarity. Correlations with significant values (**^*^**, *p* < 0.05; **^**^**, *p* < 0.01) are shown in bold number.

|  | Latitude | pH | TC | TN | C/N | H_2_O% | TP | AK | AP | NH_4_^+^-N | NO_3_^-^-N | PNR |
| --- | --- | --- | --- | --- | --- | --- | --- | --- | --- | --- | --- | --- |
| *AOA* | | | | | | | | | | | | |
| Richness | -0.345 | -0.117 | -0.086 | -0.172 | 0.384 | -0.044 | -0.238 | -0.044 | -0.172 | -0.091 | -0.092 | -0.197 |
| Diversity | -0.334 | -0.045 | -0.068 | -0.140 | 0.322 | -0.030 | -0.231 | -0.073 | -0.195 | -0.117 | -0.075 | -0.163 |
| Abundance | **0.578**** | 0.312 | **0.830**** | **0.794**** | 0.205 | **0.663**** | **0.492*** | 0.373 | -0.035 | -0.027 | -0.119 | **0.392*** |
| *AOB* | | | | | | | | | | | | |
| Richness | -0.060 | **0.515**** | 0.372 | 0.377 | 0.035 | 0.310 | **0.425*** | **0.548**** | 0.070 | -0.042 | -0.106 | **0.553**** |
| Diversity | 0.021 | **0.491*** | **0.532**** | **0.532**** | 0.075 | **0.469*** | **0.466*** | **0.572**** | 0.088 | 0.036 | -0.033 | **0.601**** |
| Abundance | 0.004 | **0.502**** | 0.170 | 0.128 | 0.165 | 0.188 | 0.167 | **0.560**** | **0.526**** | **0.524**** | **0.458*** | **0.666**** |
