# Supplementary material for: Ammonia-Oxidizing Archaea Show More Distinct Biogeographic Distribution Patterns than Ammonia-Oxidizing Bacteria across the Black Soil Zone of Northeast China
Source: Front Microbiol. 2018 Feb 9;9:171. doi: 10.3389/fmicb.2018.00171 (PMC5819564; doi:10.3389/fmicb.2018.00171)
Supplement: Table S7 — The correlations (r) and significance (p) determined by Mantel tests between community structure and soil physicochemical properties and potential nitrification rates. Correlations with significant values are shown in bold number. [file Table7.DOCX]

**TABLE S7** The correlations (r) and significance (*p*) determined by Mantel tests between community structure and soil physicochemical properties and potential nitrification rates. Correlations with significant values are shown in bold number.

|  | Mantel test | Latitude | pH | TC | TN | H_2_O% | TP | AK | AP | NH_4_^+^-N | NO_3_^-^-N | PNR |
| --- | --- | --- | --- | --- | --- | --- | --- | --- | --- | --- | --- | --- |
| *AOA* | r | **0.386** | **0.353** | **0.281** | 0.230 | 0.178 | **0.225** | 0.043 | 0.154 | -0.05 | -0.02 | 0.183 |
|  | *p* | 0.001 | 0.002 | 0.014 | 0.052 | 0.095 | 0.019 | 0.339 | 0.102 | 0.512 | 0.410 | 0.086 |
| *AOB* | r | **0.160** | **0.387** | **0.241** | **0.220** | **0.250** | **0.179** | 0.145 | -0.051 | 0.092 | 0.159 | 0.106 |
|  | *p* | 0.021 | 0.001 | 0.041 | 0.038 | 0.022 | 0.020 | 0.098 | 0.668 | 0.202 | 0.111 | 0.151 |
