# Supplementary material for: Ammonia-Oxidizing Archaea Show More Distinct Biogeographic Distribution Patterns than Ammonia-Oxidizing Bacteria across the Black Soil Zone of Northeast China
Source: Front Microbiol. 2018 Feb 9;9:171. doi: 10.3389/fmicb.2018.00171 (PMC5819564; doi:10.3389/fmicb.2018.00171)
Supplement: Table S8 — Mantel and Partial Mantel tests were conducted to compare the relative impact of spatial distance and environmental dissimilarity distance on the AOA and AOB communities. [file Table8.DOCX]

|  | Mantel | Partial Mantel |
| --- | --- | --- |
| *AOA* |  |  |
| Env | **0.24** | 0.09 |
| Distance | **0.39** | **0.32** |
| *AOB* |  |  |
| Env | **0.25** | **0.21** |
| Distance | **0.16** | 0.06 |

**TABLE S8** Mantel and Partial Mantel tests were conducted to compare the relative impact of spatial distance and environmental dissimilarity distance on the AOA and AOB communities.
